# Health Apps Require Co-development to Be Acceptable and Effective

**DOI:** 10.3389/fpsyg.2021.714453

**Published:** 2021-07-16

**Authors:** Elizabeth Ball, Carol Rivas

**Affiliations:** ^1^Department of Obstetrics and Gynaecology, Barts Health NHS Trust, London, United Kingdom; ^2^Women's Health Research Unit, Yvonne Carter Building, Queen Mary University of London, London, United Kingdom; ^3^Centre for Maternal and Child Health Research, School of Health Sciences, City University of London, London, United Kingdom; ^4^Social Research Institute, University College London, London, United Kingdom

**Keywords:** health applications, telehealth, co-development, mindfulness, app adherence, chronic pelvic pain

## Background

Mobile health apps (HA) are enjoying increasing popularity among the general population and health care providers (Kao and Liebovitz, 2017). Since they can be downloaded onto a smartphone, they can be accessed outside the house, for instance during a commute, making them convenient to use and “always there.”

HA may monitor health functions, teach, and coach users through exercise regimes, meditation or lifestyle changes, such as diet changes (Flores Mateo et al., 2015) or smoking cessation (Whittaker et al., 2019). If these are used regularly, positive habits can be formed. Even before the pandemic HA use increased further, with 165,000 mobile HAs publicly available from app stores (Sekhon et al., 2018) and use has increased since the pandemic began. For example, ORCHA, an independent digital health assessment and distribution organization reported a 25% increase in HA downloads during the COVID 19 pandemic (mainly mental health, fitness, diabetes, and weight loss)^1^. Concerns have been voiced about the lack of regulatory oversight and issues of privacy and security (Kao and Liebovitz, 2017). Moreover, the evidence base for effectiveness and usability is patchy, with research HAs being tested for efficacy but not commercially available and commercial HA not rigorously tested (de la Vega and Miró, 2014), while there has been an effort to change this recently (Göransson et al., 2018; Jensen et al., 2019; Ball et al., 2020; Forbes et al., 2020).

Pandemic and post-pandemic changes to health service delivery include a move away from face-to-face toward virtual appointments some of which may remain in the longer term (Ball, Khan submitted for publication). This trend brings into focus virtual instructions for self-management and lifestyle changes for service users: Such interventions can easily be delivered in the format of a smartphone HA, to which a service user could be signposted during a virtual appointment. HAs that teach and monitor lifestyles changes are potentially highly attractive as they are cost effective compared to face-to-face delivery and save service users money and time through reduced travel, even without pandemic constraints. They are particularly attractive given there remain Covid-19 related limitations to face-to-face meetings at the time of writing.

Medical staff at risk of poor mental health and burnout after the response to the Covid-19 pandemic are thought to benefit as well from using HA. The BartsHealth Trust website points hospital staff to the HAs “Calm©” and “Headspace©,” which both teach mindfulness meditation, remind the user to practice and chart adherence to the HA.

Since health providers are currently stretched by the backlog of work resulting from the impacts of the Covid-19 pandemic, not much time and funds are available for teaching self-management techniques to staff and service users. Therefore, it is tempting to signpost service users and staff to existing HAs, which are relatively cheap to deliver and “oven ready.”

## Researching a Mindfulness Meditation APP

The authors have experience in researching the use of an existing mindfulness meditation app (Headspace©) in women suffering from chronic pelvic pain (CPP) (Ball et al., 2020; Forbes et al., 2020). Headspace© remains one of the most favored and recommended HA by health care professionals in 2020–2021^1^. Given the benefits of using mindfulness in pain (Ball et al., 2017), it was hypothesized this would also benefit women with CPP.

### Methodology

To study the efficacy and acceptability of app-delivered mindfulness meditation in CPP in women we carried out the Memphis trial, a three-arm randomized controlled trial (*n* = 90, in a 1:1:1 ratio) with user-recoded health and usability outcomes via postal questionnaires or telephone at 2-, 3- and 6-months post-randomization. Additional qualitative pre- and post-study interviews of women with CPP and health care professionals were carried out to gain a deeper understanding of obstacles and facilitators to app use.

The intervention studied was a new CPP module, developed by the Headspace© author, Andy Puddicombe, which was added to the existing Headspace platform, the other arms were app-delivered instructions for progressive muscle relaxation and treatment as usual. Both app-delivered arms were accessible for 60 days.

### Findings and Discussion

The pre-study design advisory group made up of highly motivated young educated women from the areas where the trial was planned liked the app a lot and used it frequently during the run up to the meeting.

The actual study participants were young women (mean 35 years) recruited from the same two areas of London with high levels of deprivation and a large range of ethnic diversity. However, findings differed from what was anticipated from the focus group: Reasons for this could include that the focus group comprised of self-selected women, who had more time than the average study participant, given that they were able to attend an evening focus group (lack of time was given a reason not to use the HA) and more motivated toward self-care, such as using a HA. Social and educational factors could have also played a role. The advisory group had comprised white ethnic minority women, whereas 64% of women in the trial intervention arm were Black or Asian. The app use was too low for a meaningful analysis of its efficacy (Forbes et al., 2020) but qualitative interviews revealed an insight into reasons for low app usage. Themes included issues of familiarity and capabilities with app technology, motivations to using the app, perceived benefits, relation to other therapies and opportunities to use the app (technology issues getting in the way, life getting in the way).

When technical difficulties appeared that could not be resolved with the help of family it led to abandoning of the app or restricted use of its functionality, as recalled by one participant: “I am not good with technical things … I consulted with my daughter and she helped me work it out… so I don't try everything.” The need for additional support in the form of face-to-face instruction or manuals/YouTube videos was highlighted: “If your market is targeting people who are not using apps then you are going to have to get together and find ways to do this,” as one participant said. The Headspace app requires regular use to learn and benefit from psychological techniques and has user-set reminder alarms. However, some participants used it only during pain exacerbations and did not really appreciate the way it should be used for maximum benefit. Others blamed underusing the app on having no spare time because of juggling work and children. Health care professionals were concerned that to face-to-face instructions would compound effective use of staff contact time with patients and cancel out the savings hoped for through the HA. Despite general under-use of the app some users felt a subjective positive impact on their lives. One participant, who felt empowered by using the app stated: “I think it was day 3, I could see the change that was happening, I was able to speak up for myself ……I can't explain it, even now I am getting emotional… it's just a lack of focus, I just needed direction.”

The Memphis study highlighted, that patients did not only lack familiarity with apps, they also met practical issues in downloading the app in the first place which also took up staff time. Staff pointed out that not all patients had smartphones and some patients lacked the storage space to load the app on their phones. There were also issues with Wi-Fi connectivity when staff tried to help the patients load the app within the hospital sites. Possible solutions that staff suggested were to lend patients phones with the app already downloaded, or to have group download sessions in a location with good Wi-Fi signal, though they acknowledged the cost implications.

Other studies that have been carried out on the same Headspace© HA without the CPP module showed low rates of dropout, particularly when people were taken out of their day-to-day routine and had extra time, for instance in a psychiatric inpatient unit (Mistler et al., 2017).

Young urban educated participants (Laurie and Blandford, 2016), had a low dropout rate but still experienced challenges incorporating the HA into a busy lifestyle.

## The Way Forward

The neglected topic surrounding challenges in the governance and development of HA have been discussed (Magrabi et al., 2019). The authors are concerned that “while a bottom-up approach is advantageous for innovation and clinical engagement, apps developed in this manner may not be sustainable beyond pilot testing.” They acknowledge “the diverse contexts in which HA are developed and the heterogeneity of the users, including varying levels of health literacy and IT skills.” Users may not “have the skills to use an app in the manner was intended by the designers.” This resonates with our experience.

Despite the undeniable benefits of HA they are certainly not a panacea or replacement for face-to-face interventions. We recognize the phenomenon of “zoom fatigue” ascribed to factors such as reduced mobility and high cognitive load from navigating technology during the remote virtual meetings (Bailenson, 2021). This could fuel a desire for face-to-face human interaction.

A blended program combining HAs with “human factor” input to remain motivated, showing commitment to a person to continue a program could be a promising approach and was suggested by participants in our study (Ball et al., 2020). Some users may require more technical support and input, others more reminders or gamification to give attention to regular app practice. Gamification elements such as goal setting, providing feedback on performance, reinforcement, comparing progress and social connectivity are variants of techniques associated with changes of health behavior.

We cannot assume that all “digital natives” are comfortable installing and navigating apps. We are particularly concerned about service users from deprived backgrounds. Hurdles to HA and, more widely, virtual medicine use, including remote consultations are multi-fold: Obstacles to accessing phone and video appointments for these women include language barriers, access to phone/video technology, familiarity and acceptance of virtual activities, lack of privacy due to domestic overcrowding and lack of phone ownership. Other important obstacles are connectivity and access to WIFI broadband.

A recent scoping search on HA concluded that physicians should play an active role in recommending and supervising health app use to reach digital-illiterate or health-illiterate people (Heidel and Hagist, 2020). We acknowledge that the evaluation of an existing app is often appropriate (Boudreaux et al., 2014) and is both quicker and more cost-effective than designing an app from scratch, but wish to share the learning points from our experience. We believe HA can make a valuable contribution to health care delivery but “oven ready” solutions may not fit all and co-developing HA from the start with the envisaged service users (Mrklas et al., 2020) may lead to higher uptake. This requires creative strategies when including seldom heard service users, such as recruiting from a helpline providing help to those who struggle with virtual interventions. The so-called BAME (Black and Asian minority ethnic minorities) Toolkit developed by the Centre for BME Health in Leicester^2^ is a useful resource to help address issues of inclusion and equality with respect to BAME communities. Edwards et al. (2016) emphasize the importance of involving a collaboration of app developers, behavioral scientists and public health practitioners to co-develop HA, to this we would add the importance of including feedback from patients that is fully representative of the target group.

## Author Contributions

EB and CR were principal and co-investigators on the MEMPHIS trial. EB wrote the article with editorial input and oversight from CR. Both authors contributed to the article and approved the submitted version.

## Conflict of Interest

The authors declare that the research was conducted in the absence of any commercial or financial relationships that could be construed as a potential conflict of interest.

## Footnotes

^1^https://www.orcha.co.uk/media/1746/covid_report_jan_2021_final-version.pdf

^2^https://arc-em.nihr.ac.uk/clahrcs-store/increasing-participation-black-asian-and-minority-ethnic-bame-groups-health-and-social.
